# Monoethanolamine-induced glucose deprivation promotes apoptosis through metabolic rewiring in prostate cancer

**DOI:** 10.7150/thno.62724

**Published:** 2021-08-27

**Authors:** Chakravarthy Garlapati, Shriya Joshi, Ravi Chakra Turaga, Manjari Mishra, Michelle D Reid, Shobhna Kapoor, Liana Artinian, Vincent Rehder, Ritu Aneja

**Affiliations:** 1 Department of Biology, Georgia State University, Atlanta, GA, USA-30303; 2 Caris Life Sciences, Tempe, Arizona, USA-85282; 3 Department of Chemistry, Indian Institute of Technology Bombay, Powai, Mumbai, India-400076; 4 Department of Pathology & Laboratory Medicine, Emory University School of Medicine, Atlanta, GA, USA-30324

**Keywords:** prostate cancer, monoethanolamine, metabolism, autophagy, apoptosis

## Abstract

**Rationale**: Cancer cells rely on glucose metabolism for fulfilling their high energy demands. We previously reported that monoethanolamine (Etn), an orally deliverable lipid formulation, reduced intracellular glucose and glutamine levels in prostate cancer (PCa). Glucose deprivation upon Etn treatment exacerbated metabolic stress in PCa, thereby enhancing cell death. Moreover, Etn was potent in inhibiting tumor growth in a PCa xenograft model. However, the precise mechanisms underlying Etn-induced metabolic stress in PCa remain elusive. The purpose of the present study was to elucidate the mechanisms contributing to Etn-mediated metabolic rewiring in PCa.

**Methods**: Glucose transporters (GLUTs) facilitate glucose transport across the plasma membrane. Thus, we assessed the expression of GLUTs and the internalization of GLUT1 in PCa. We also evaluated the effects of Etn on membrane dynamics, mitochondrial structure and function, lipid droplet density, autophagy, and apoptosis in PCa cells.

**Results**: Compared to other GLUTs, GLUT1 was highly upregulated in PCa. We observed enhanced GLUT1 internalization, altered membrane dynamics, and perturbed mitochondrial structure and function upon Etn treatment. Etn-induced bioenergetic stress enhanced lipolysis, decreased lipid droplet density, promoted accumulation of autophagosomes, and increased apoptosis.

**Conclusion**: We provide the first evidence that Etn alters GLUT1 trafficking leading to metabolic stress in PCa. By upregulating phosphatidylethanolamine (PE), Etn modulates membrane fluidity and affects mitochondrial structure and function. Etn also induces autophagy in PCa cells, thereby promoting apoptosis. These data strongly suggest that Etn rewires cellular bioenergetics and could serve as a promising anticancer agent for PCa.

## Introduction

Recent advancements in cancer therapies have led to increasing interest in more effective and less toxic lipid-based drugs that can target metabolic pathways 1. One such lipid-based formulation, phosphoethanolamine (PhosE) 2, 3, recently garnered significant attention in Brazil and has become a subject of intense cancer research 4, 5. In a recent study, we demonstrated that monoethanolamine (Etn), an orally deliverable lipid precursor, could suppress prostate cancer (PCa) growth 6. In contrast to conventional chemotherapies, Etn modulates energy and metabolic sources, such as glucose and glutamine. In addition to its potent anticancer activity, Etn is cost-effective and easily soluble, and exhibits excellent pharmacokinetic and toxicokinetic properties 6. Thus, Etn may serve as a promising PCa drug regimen.

Metabolic reprogramming has been recognized as one of the hallmarks of cancer, allowing highly proliferative cancer cells to meet their high bioenergetic, biosynthetic, and redox demands 7-9. Metabolic reprogramming in cancer cells includes increased glucose uptake and enhanced glutaminolysis and fatty acid synthesis; these metabolic shifts are vital for the sustained growth and survival of tumor cells 10. Glucose transport across the plasma membrane (PM) is the first and rate-limiting step of glucose metabolism in mammalian cells and is mediated by glucose transporter (GLUT) proteins. High glucose uptake in malignant cells is associated with increased expression of GLUTs 11. Several mechanisms of metabolic rewiring, including alterations in glutaminolysis, glycolysis, lipid metabolism, and mitochondrial biogenesis, have been identified thus far 7, 9. Mitochondria play a central role in this process by synthesizing molecules vital for cancer cell proliferation, survival, and metastasis 12, 13. As lipids are an essential energy source when other nutrients are exhausted, cancer cells rely on lipid metabolism to meet their high energy requirements 14, 15. Thus, cancer cells accumulate lipid droplets (LDs), which serve as reservoirs of saturated lipids and cholesteryl esters, supporting tumor cell survival and growth 9, 15, 16. Hence, targeting metabolic reprogramming in cancer cells has emerged as a promising approach to reduce the tumor burden. Over the years, many compounds have been shown to selectively target metabolic enzymes imperative for tumor growth and neoplastic progression; however, the clinical success of these compounds is limited 7.

We have previously shown that Etn is a promising candidate drug to target metabolic reprogramming in PCa 6. Etn is converted to phosphatidylethanolamine (PE) via the Kennedy pathway through ATP and CTP-dependent phosphorylation. The other substrate involved in the Kennedy pathway is choline, which is converted to phosphatidylcholine (PC) 17. PE and PC are the most abundant glycerophospholipids in cells and are enriched in the inner leaflet of the PM and the cristae of the inner mitochondrial (IM) membranes 18. PE is required for the activity of several respiratory complexes and plays a crucial role in autophagy induction 18. The balance between different phospholipids is critical for maintaining cellular metabolism and homeostasis 19, 20. Neoplastic transformation is accompanied by alterations in the turnover of PE and other phospholipids, such as PC 21, 22. Aberrant PC deacylation to glycerophosphocholine (GPC) can lead to apoptosis, and the PC/GPC ratio has been proposed as a means to monitor treatment response in cancer patients, where a decreased PC/GPC ratio has been associated with favorable survival outcomes 23-27. Similar to PC, PE is deacylated to glycerophosphoethanolamine (GPE) 27. Although Etn is known to modulate metabolism and suppress PCa growth 6, the relevance of Etn and PE/GPE ratio in inducing metabolic stress in PCa remains elusive.

In this study, we investigated the effect of Etn on the PE/GPE ratio and mitochondrial structure and function in PCa cells. We also assessed the role of Etn in cellular bioenergetics and survival in PCa. Herein, we provide evidence that Etn induces metabolic stress in PCa cells by modulating cellular bioenergetics at various levels, ultimately leading to apoptosis. Thus, Etn may serve as an effective treatment for PCa.

## Methods

### Cell culture

PC3 and DU-145 cells were purchased from the Perkin Elmer and American Type Culture Collection, respectively. Cells were cultured in EMEM, or RPMI medium (Corning) supplemented with 10% fetal bovine serum (HyClone, Canadian origin). Cells were maintained at 37 °C in a humified atmosphere containing 5% CO_2_.

### Etn treatment

Cells were seeded in cell culture dishes as per the experimental requirements and were allowed to reach 60%-70% confluency. The next day, cells were treated with 0.75 mg/mL cell culture grade Etn (Sigma-Aldrich, #E0135) for 1, 4, 24, and 48 h at pH 7.4. Untreated cells served as controls.

### Patients' samples

Formalin-fixed paraffin-embedded (FFPE) tissue samples from patients with PCa were obtained from Emory hospital, Atlanta, USA, with prior patient consent. Samples were classified according to the tubular differentiation pattern as high grade (n = 26), low grade (n = 12), or benign (n = 13). The differentiation patterns were confirmed by a pathologist using hematoxylin-eosin (H&E) staining. Adjacent normal prostate FFPE samples (n = 13) were collected for comparison.

### Immunohistochemistry and specimen scoring

FFPE samples were sectioned (5 µm) for immunohistochemistry (IHC). Tissues deparaffinized and rehydrated in serial ethanol solutions (100%, 95%, 70%, 50%) were placed in citrate buffer (pH 6.0) in a pressure cooker at 15 psi for 10 min for antigen retrieval. GLUT1 was immunostained using a rabbit monoclonal GLUT1 antibody (Abcam, #ab115730; 1:250). Enzymatic detection of the antibody was performed using Betazoid DAB Chromogen Kit (BioCare Medical, #BDB2004). Stained tissues were reviewed and scored by a pathologist who was blinded to the clinical annotations. Scoring was performed for both the intensity of staining (0, none; 1, low; 2, moderate; 3, high) and the percentage of positive cancer cells expressing GLUT1 (0, < 1%; 1, 1-24%; 2, 25-49%; 3, 50-74%; 4, 75-100%); the combined scores were recorded.

### Glucose uptake assay

The glucose uptake assay in PCa cells with and without Etn treatment was performed according to the manufacturer's instructions (Promega, #J1342). Briefly, ~5000 PC3 and DU-145 cells were seeded in 96-well plates. Cells at 60%-70% confluency were treated with Etn at different time points (1 h and 48 h); in control cells, the cell culture medium was changed at the respective time points. Cells were treated with the reagents provided in the kit according to the manufacturer's guidelines. Finally, 2-deoxyglucose-6-phosphate (2DG6P) concentration proportional to the luminescence was recorded using a luminescence plate reader. A standard curve was created to calculate the concentration of 2DG6P relative to the luminescence signal.

### Real-time PCR

mRNA from untreated and Etn-treated PCa cells was extracted using the RNeasy kit (Qiagen) and quantified using NanoDrop. cDNA was prepared using an iScript cDNA synthesis kit (Bio-Rad, #1708891) as per the manufacturer's instructions. cDNA quality was confirmed by agarose gel electrophoresis. Real-time PCR (RT-PCR) reactions were prepared in triplicates using SsoAdvanced Universal SYBR Green Supermix (Bio-Rad, #1725271). Custom RT-PCR primers designed and validated using Primer3Plus were synthesized by Sigma-Aldrich. RT-PCR was performed using 7500 Real-Time PCR System (Applied Biosystems), and expression data were analyzed using 7500 FAST software v2.0.6. Data were normalized to the mRNA levels of β-actin. The following primers were used:

GLUT1: forward, 5'-CCAGCAGCAAGAAGCTGACG-3'; reverse 5'-CCATAGCGGTGGACCCATGT-3'

GLUT3: forward, 5'-GAGACCCGTGGCAGGACTTT-3'; reverse 5'-AGCAGGCTCGATGCTGTTCA-3'

GLUT4: forward, 5'-CAGTGCTACCTGCCCTCCTG-3'; reverse 5'-ATCCTTCAGCTCAGCCAGCA-3'

GLUT11: forward, 5'-CCCTCATCGTGTCTCTGTATCC-3'; reverse 5'-CGAGCAGTCTTCCCAGCAT-3'

β-actin: forward, 5'-CTGGAACGGTGAAGGTGACA-3'; reverse 5'-AAGGGACTTCCTGTAACAATGCA-3'

### Filipin staining

PCa cells were seeded on 22 × 22 mm coverslips in a 6-well plate. Cells at 60%-70% confluency were treated with Etn (1 h, 48 h). Untreated cells were used as controls. Subsequently, cells were fixed in 3% paraformaldehyde for 1 h at room temperature (RT) and then washed with phosphate-buffered saline (PBS). Coverslips were stained with Filipin III solution (0.05 mg/mL; Cayman chemical, #704401) for 2 h at RT. Coverslips were mounted in ProLong Gold Antifade Reagent (Invitrogen, #P36930) and observed under a fluorescence microscope using a UV filter (340-380 nm excitation). Quantification analysis was performed in images acquired from five random regions of the slides using ImageJ's line plot profile function tool.

### Measurement of oxygen consumption rate and extracellular acidification rate

Oxygen consumption rate (OCR) and extracellular acidification rate (ECAR) were measured using a Seahorse XF96 extracellular flux analyzer, as previously described 28. Briefly, 2 × 10^4^ PCa cells were seeded in a Seahorse XF96 plate. After 24 h of incubation, samples were subjected to Seahorse XF96 extracellular flux analysis, and cell treatments were performed in the following sequence: Etn, oligomycin, carbonyl cyanide-4 (trifluoromethoxy) phenylhydrazone (FCCP), and rotenone/antimycin. OCR and ECAR data were analyzed using Wave 2.6.0 software (Agilent Technologies).

### Live-cell imaging of mitochondrial membrane potential

Cells were treated with Etn for different time intervals. Subsequently, cells were incubated with rhodamine 123 (Sigma-Aldrich, #R8004) for 1 h at 37 °C. Cells were washed with PBS and analyzed by flow cytometry (excitation wavelength: 488 nm; emission wavelength: 515-575 nm). After the acquisition of 10000 events, the side scatter (SSC) was plotted against the forward scatter (FSC). Appropriate gating was performed to select positive cells while excluding debris and doublets. Flow cytometry data were analyzed using FACSDiva software v9.0 (BD Biosciences).

### Transmission electron microscopy

PCa cells were seeded in 12-well plates followed by Etn treatment for 1 h and 48 h. Transmission electron microscopy (TEM) was carried out as previously described 29. Briefly, cells were fixed with 2.5% glutaraldehyde in 0.1 M cacodylate buffer (pH 7.4) and washed twice with 0.1 M cacodylate buffer (pH 7.4). Cells were then fixed with 1% osmium tetroxide for 1 h. After washing with cacodylate buffer, cells were dehydrated in graded ethanol. Cells were infiltrated overnight with a 50:50 mixture of 100% ethanol and Eponate 12 resin (Ted Pella, Inc.). This step was repeated once again, followed by an additional change with 100% Eponate 12 resin. Cell monolayers embedded in multi-well plates were incubated at 60 °C for polymerization. Ultrathin sections (70-80 nm) were prepared using a Leica EM UC6 ultramicrotome, and sections were placed on 200 mesh copper grids for TEM. Sections were then stained with 5% uranyl acetate for 15 min, followed by incubation with 2% lead citrate for 15 min. Images were acquired using a JEOL JEM-1400 TEM (Tokyo, Japan) equipped with a Gatan US1000 CCD camera (Pleasanton, CA). Mitochondria and autophagosomes were manually counted from five random fields using the cell counter plugin of ImageJ.

### Membrane fluidity analysis by two-photon microscopy

PCa cells were seeded on coverslips in 6-well plates. After Etn treatment, cells were incubated with 5 µM Laurdan (6-lauryl-2-dimethylamino-naphthalene; Invitrogen, #D250) for 30 min at 37 °C. After PBS washes, cells were fixed with 4% paraformaldehyde for 15 min at 37 °C in a 5% CO_2_ incubator. Coverslips were mounted onto glass slides, and images were acquired using a two-photon Leica SP8 microscope in the λ mode as described previously 30. Images were analyzed using the spectral imaging toolbox 31.

### Ultra-high-pressure liquid chromatography-mass spectrometry

Phospholipids extracted from Etn-treated and control cells using the Bligh-Dryer method 32 were dried under nitrogen purge. Samples were submitted to Thermo Fisher Scientific for lipidomic and metabolomic analysis.

### Mouse xenograft models

Nude (nu/nu; stock no: 002019) male mice were purchased from the Jackson Laboratory. All animal experiments strictly adhered to institutional animal care and use committee (IACUC) guidelines. Power analysis using the G power software was performed to determine the number of animals required for this study. PC3 cells (~5 × 10^6^) were subcutaneously injected into the right flanks of mice. When tumors reached 100 mm^3^, mice were divided into two groups: vehicle and Etn treatment. Etn (40 mg/kg; pH 7.4, adjusted using phosphoric acid) was administered to mice every day for 28 days (4 weeks) using oral gavage. PBS was administered orally to mice in the vehicle group. Tumor growth was monitored using vernier calipers (twice/week or every three days), and body weight was recorded for up to 4 weeks. Tumor volume was calculated using the formula length × (width)^2^/2. At the end of the experiment, all mice were euthanized following the IACUC guidelines, and tumors from vehicle and Etn-treated mice were collected. Tumors were divided into two parts; one part was fixed in 10% formalin, and the other part was stored in liquid nitrogen. FFPE blocks prepared from formalin-fixed tissues were sectioned (5 µm).

### Oil Red-O staining

PCa cells seeded on coverslips in 6-well plates were treated with Etn for different time points. Cells were fixed in 10% formalin for 30 min, followed by 5 min incubation with 60% isopropanol. LDs were stained with Oil Red-O staining (ORO) dye (Sigma-Aldrich, #O0625), and cell nuclei were counterstained with hematoxylin (Agilent Technologies, #CS700). Coverslips were mounted with Aquatex (Millipore, #108562) and imaged under a light microscope equipped with a Fein Optic 3DCxM12 camera. Densitometry analysis was performed using ten images per time point. Images were color-thresholded using ImageJ to select for the stained areas. The percentage of stained areas was calculated as follows: % ORO = (red area) / (total occupied area) × 100.

### Lipolysis assay

PCa cells were seeded in 12-well plates followed by overnight incubation at 37 °C in a humidified 5% CO_2_ incubator. Etn-treated and untreated control cells were incubated for different time points. After treatment, the cell culture media was collected and analyzed for glycerol concentration using the Free Glycerol Determination kit (Sigma-Aldrich, #FG0100) according to the manufacturer's instructions. Colorimetric measurements were performed at 570 nm. A standard curve was used to determine the concentration of glycerol.

### YO-PRO 1 and propidium iodide apoptosis assay

Cells seeded in 70-mm cell culture plates were treated with chloroquine (CQ; Sigma-Aldrich, #C6628), 3-methyladenine (3MA; Sigma-Aldrich, #M9281), and Etn for different time points. UV-irradiated cells served as a positive control. Cells were harvested and incubated with 1 µL YO-PRO-1 (100 nM final concentration; Invitrogen, #Y3603) and 1 µL propidium iodide (PI; 1 µg/mL final concentration) on ice for 20-30 min. Stained cells were analyzed using flow cytometry. Compensation was performed using single-color stained samples. The percentage of apoptotic cells was calculated using FlowJo.

### Immunoblotting

PCa cells seeded in 100-mm cell culture dishes were treated with Etn for different time points. After treatment, cells were subjected to immunoblotting as described previously 33. Lysates from fresh tumor samples were prepared using BeadBlaster microtube homogenizer (LabRepCo) following the manufacturer's guidelines. Proteins were separated by sodium dodecyl sulfate-polyacrylamide gel electrophoresis (SDS-PAGE) and then were transferred onto polyvinylidene difluoride (PVDF) membranes. Membranes were incubated with anti-light chain 3 primary antibody (LC3; Cell Signaling Technologies, #2775S, 1:500) or GLUT1 primary antibody (Abcam, #ab115730, 1:1000) overnight at 4 °C; anti-beta-actin primary antibody (Santa Cruz Biotechnology, #SC-47778, 1:1000) was used as a loading control. Subsequently, membranes were incubated with goat anti-mouse (Santa Cruz Biotechnology, #sc-516102, 1;8000) or goat anti-rabbit (Santa Cruz Biotechnology, #sc-2357, 1:8000) IgG horseradish peroxidase-conjugated secondary antibodies. All antibodies against pro-apoptotic proteins were from Pro-Apoptosis Bcl-2 Family Antibody Sampler Kit (Cell Signaling Technologies, #9942T). The signal was visualized using the Pierce ECL Western Blotting Substrate (Thermo Fisher Scientific, #32106). Signal intensities were quantified using ImageJ and were normalized to the respective β-actin signal intensity.

### Immunofluorescence staining and confocal microscopy

Cells seeded on coverslips in 6-well plates were allowed to reach 80% confluency. Subsequently, cells were treated with Etn for different time points. After PBS washes, cells were fixed with formaldehyde for 30 min at -20 °C. Cells were thoroughly washed with PBS and blocked using 5% bovine serum albumin in 0.1% Triton X-100 for 1 h. After blocking, cells were incubated with anti-GLUT1 antibody (Abcam, #ab115730, 1:400) for 45 min at 37 °C. Cells were then incubated with a fluorescently labeled (Alexa Fluor 488) goat anti-rabbit secondary antibody (Invitrogen, #A-11034, 1:2500) for 45 min 37 °C. Nuclei were counterstained with Hoechst 33342 (Invitrogen, #H3570, 1:12000). Coverslips were mounted using ProLong Gold Antifade (Invitrogen, #P36930) and imaged using an LSM 700 confocal microscope. Quantification analysis was performed on images acquired from five random regions of the slides using the line plot profile function tool of ImageJ.

### GFP-LC3 fluorescence microscopy

PCa cells (~7.5 x 10^4^) were seeded on coverslips and transiently transfected with a puro-GFP-LC3 plasmid (kindly gifted by Prof. Anirban Banerjee, IIT Bombay) using Lipofectamine-3000 (Thermo Fisher Scientific, #L3000001) for 24 h according to the manufacturer's instructions. Cells treated with the mTOR inhibitor rapamycin (20 nM) served as a positive control to confirm the formation of LC3-GFP puncta. Transfected cells were treated with Etn at 37 °C and 5% CO_2_. After treatment, cells were fixed with 4% paraformaldehyde for 15 min at RT and rinsed with 1X PBS. Images were taken on LSM (Carl Zeiss) using 63X magnification. All post-acquisition images were used for GFP-LC3 (puncta) quantification using ImageJ (ITCN plugin). Quantification was performed by dividing the number of cells with GFP-LC3 puncta by the total number of GFP-positive cells. A minimum of 70 cells (per condition and experiment) from randomly selected fields was scored.

### Subcellular membrane fractionation

PCa cells seeded in 100-mm plates were treated with Etn for 1 h and 48 h. Cells were trypsinized and washed with ice-cold PBS. Cell lysis was performed using the subcellular protein fractionation kit (Thermo Fisher Scientific, #78840) following the manufacturer's protocol. After separating the membrane and cytosolic fractions, lysates were subjected to immunoblotting to evaluate GLUT1 (Abcam, #ab115730, 1:1000) and PKC (Abcam, #ab179522, 1:1000) levels. β-actin and EGFR (CST, #4267S, 1:1000) served as loading controls for cytosolic and membrane fractions, respectively.

### Statistical analysis

All experiments were performed thrice, and data from all experiments were used to calculate statistical significance. Statistical significance was determined using a two-tailed Student's *t*-test with Welch's correction or two-way analysis of variance (ANOVA) with Tukey's test for multiple comparisons. Data were expressed as mean ± standard error mean (SEM). Statistical analyses were performed using GraphPad Prism 9.

## Results

### GLUT1 is upregulated in PCa in a grade-dependent manner

**Figure 1:**
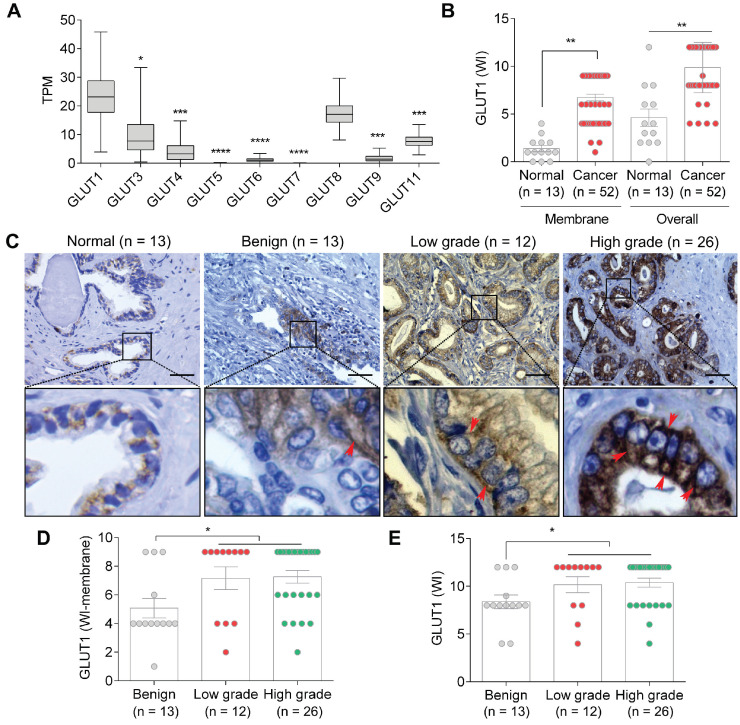
** GLUT1 expression in PCa. (A)** GLUT1 expression levels are significantly higher than those of GLUT3-9 and 11 (*n* = 497). **(B-C)** Membrane and overall GLUT1 levels in normal vs. PCa tissues **(B)**, and representative IHC images **(C)** in normal prostate, benign, low-grade, and high-grade PCa tissues; red arrows indicate membrane staining. **(D-E)** WI of membrane **(D)** and overall **(E)** GLUT1 levels in benign, low-grade, and high-grade PCa tissues. TPM: transcripts per million. Bars indicate mean ± SEM. Unpaired two-tailed Student's *t*-test with Welch's correction was used to determine the statistical significance (*P < 0.05, **P < 0.005, ***P < 0.0005). The scale bar represents 10 µm.

Glucose is a fundamental energy source, and the fine-tuning of glucose uptake is critical for glucose homeostasis and metabolic plasticity in cancer cells 34. We have previously demonstrated that Etn promotes the depletion of cellular glucose and glutamine in PCa cells 6. As GLUTs facilitate glucose uptake 35 and GLUT1 is often overexpressed in various cancers, including PCa 36, we explored the expression levels of various GLUTs in PCa using the cancer genome atlas (TCGA) dataset and the UALCAN portal 37. TCGA analysis revealed a significant upregulation of GLUT1 compared to other GLUTs in PCa (**Figure 1A**), suggesting that GLUT1 plays a predominant role in glucose uptake in PCa cells. Consistently, higher overall and membranous staining of GLUT1 was observed in PCa samples compared with normal and benign prostate samples (Emory hospital, Atlanta, USA) (**Figure 1B-C**). GLUT1 was particularly upregulated in higher grades of PCa (**Figure 1D-E**). It has been previously shown that PCa cells have a very high glucose requirement 36; hence, GLUT1 upregulation may represent a mechanism by which PCa cells meet their high glucose demands.

### Etn induces metabolic stress by impairing GLUT1 recycling in PCa cells

**Figure 2:**
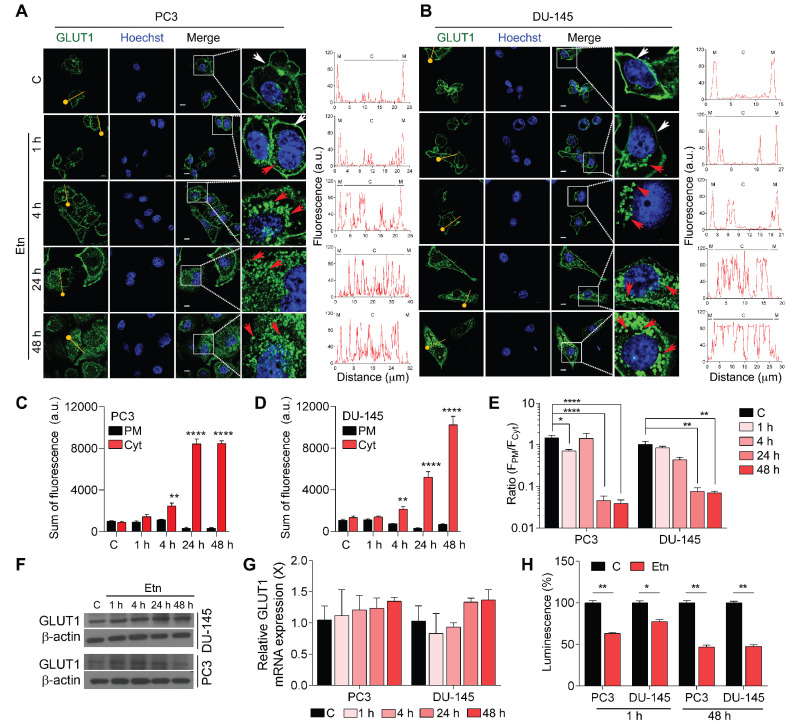
** Altered GLUT1 localization in Etn-treated PCa cells. (A-B)** Immunofluorescence staining assessing the changes in GLUT1 localization in Etn-treated PC3 **(A, left)** and DU-145 **(B, left)** cells at early and late time points. Fluorescence intensity measurements were recorded using plot profile analysis **(A-B, right)**. The yellow line indicates the line plot profile function used for measuring the fluorescence intensity. White arrows indicate membrane GLUT1, whereas red arrows indicate cytoplasmic GLUT1. **(C-D)** Quantitative bar graphs showing the sum of membrane and cytoplasmic GLUT1 fluorescence intensities in PC3 **(C)** and DU-145 **(D)** cells. **(E)** Bar graphs indicating the ratio of GLUT1 fluorescence intensity (F_PM_/F_Cyt_) in Etn-treated PCa cells. **(F)** Immunoblot showing GLUT1 levels in PCa cells treated with Etn for different time points. **(G)** *GLUT1* mRNA levels in Etn-treated PCa cells. **(H)** Glucose uptake in PCa cells with and without Etn treatment at early and late time points. a.u: arbitrary units, X: fold change, PM: plasma membrane, Cyt: cytoplasm. Bars indicate mean ± SEM. Unpaired two-tailed Student's *t*-test with Welch's correction was used to determine the statistical significance (*P < 0.05, **P < 0.005, ***P < 0.0005). The scale bar represents 5 µm.

PCa cells have been shown to exhibit elevated glycolytic flux predominantly through GLUTs 38, especially GLUT1. GLUTs are shuttled between the PM and the cytosol to load and release substrate molecules on either side of the membrane 39. Therefore, we assessed the effect of Etn treatment on GLUT1 trafficking and glucose depletion in PCa. Our data revealed an internalization of GLUT1 to the cytosol starting after 1 h of Etn treatment and becoming prominent after 48 h of treatment in both PCa cell lines (**Figure 2A-B and Figure S1A-C**). Immunofluorescence staining confirmed the effect of Etn treatment on GLUT1 shuttling between the PM and the cytosol (**Figure 2A-E**). The fluorescence intensity of GLUT1 in the PM decreased upon Etn treatment, especially during the late time points of the treatment. Concomitantly, an increase in the cytosolic GLUT1 fluorescence was observed in Etn-treated PCa cells (**Figure 2A-B**). A significant increase in the sum of fluorescence intensity in the cytoplasm and a decrease in the ratio of plasma membrane fluorescence (F_PM_) to cytoplasm fluorescence (F_Cyt_) was observed in Etn-treated PCa cells starting from 4 h of treatment (**Figure 2C-E**). However, no significant differences in GLUT1 protein or mRNA levels were observed between Etn-treated and untreated PCa cells (**Figure 2F-G, and Figure S1D**). Similarly, the expression levels of other GLUTs remained unaltered upon Etn treatment (**Figure S1E-F**). A similar effect on GLUT1 internalization (**Figure 4G-H**) and protein level (**Figure S1G-H**) was observed in PCa xenografts in mice treated with Etn. These *in vitro* and *in vivo* evidence support that rather than modulating GLUT1 expression, Etn alters GLUT1 recycling and trafficking. Upon Etn treatment, GLUT1 entering the cytosol is not effluxed back to the PM, leading to reduced levels of membrane bound GLUT1 and promoting metabolic stress in PCa. Lee et al. 40 have shown that PKC phosphorylates GLUT1 at S226 to facilitate glucose transport in primary endothelial cells. As GLUT1 phosphorylation at S226 enhances GLUT1 membrane localization. Thus, we then explored whether GLUT1 phosphorylation through PKC is responsible for impaired membrane localization after Etn treatment. However, we did not observe a significant effect of Etn on PKC localization **(Figure S1A)**. Thus, impaired membrane localization of GLUT1 upon Etn treatment is not through the PKC route. As we observed a net reduction of GLUT1 levels in the PM following Etn treatment, we next evaluated glucose uptake in Etn-treated cells. We observed a significant decrease in glucose uptake upon Etn treatment in both PCa cell lines (**Figure 2H**). Since glucose is the primary energy source for most cancer cells, a lowered glucose uptake could impair the ability of cancer cells to meet their high energy demands. Altogether, these results indicate that Etn can modulate GLUT1 trafficking, thereby depriving PCa cells of glucose.

### Etn disrupts membrane dynamics by decreasing membrane fluidity in PCa cells

**Figure 3:**
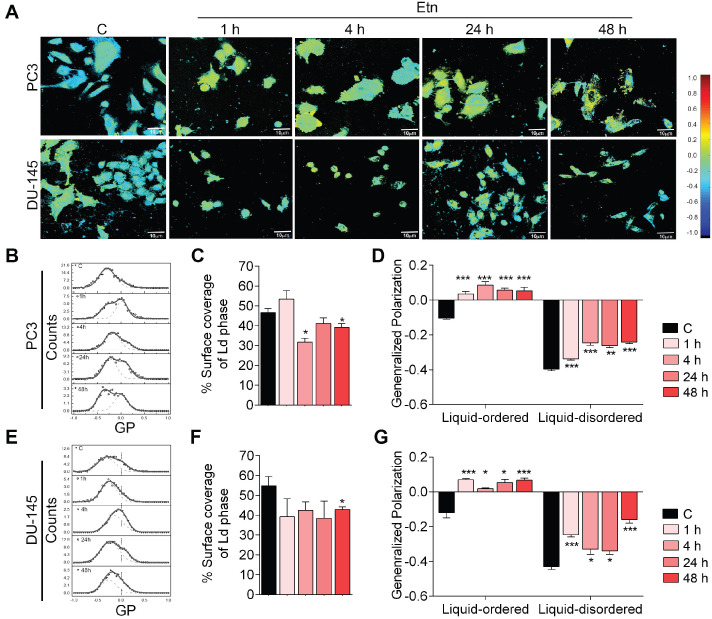
** Etn treatment decreases membrane fluidity in PCa cells. (A-B, E)** Two-photon global GP images (n = 20) **(A)** and histograms **(B, E)** deconvoluted by fitting two-Gaussian distributions; the percentage of the surface coverage of Ld phase distribution was expressed as the area under the curve **(B, E)** of the two Gaussian distributions. **(C, F)** Bar graphs showing the percentage surface coverage of the Ld phase in PCa cells. **(D, G)** Bar graphs showing the liquid-ordered and liquid disordered phases in PCa cells treated with Etn for different time points. Bars indicate mean ± SEM. Unpaired two-tailed Student's *t*-test was used to determine the statistical significance (*P < 0.05, **P < 0.005, ***P < 0.0005). C = control. The scale bar represents 10 µm.

Glucose transport across the PM through GLUTs is strongly influenced by membrane fluidity 41-43. To investigate how Etn treatment impaired GLUT1 recycling and glucose uptake, we evaluated the effect of Etn treatment on membrane fluidity and dynamics. Fluid regions of the membrane (liquid disordered, Ld) exhibit Laurdan peak emission at 490 nm, whereas less fluid regions (liquid-ordered, Lo) show peak emission at 440 nm 44, 45. The ratiometric measure of these two emission regions, known as generalized polarization (GP), provides insight into the lipid fluidity in live cell membranes. GP values between 0.25 and 0.55 indicate Lo domains, whereas Ld domains have 0.25 < GP < -0.55 44, 45. Pseudo-color GP images of untreated PCa cells showed a heterogeneous cell membrane lipid distribution (**Figure 3A**). Notably, Etn altered the membrane lipid distribution in a time-dependent manner, with a profound decrease in the overall membrane fluidity after 48 h of treatment (**Figure 3D-G**) as the surface coverage of Ld domains decreased (**Figure 3C-D**). This was accompanied by a shift in the global GP distribution to higher values (**Figure 3B, E**). The global GP histogram of untreated PCa cells exhibited a bimodal distribution centered at distinct GP values, indicating the coexistence of fluidic and ordered membrane regions (**Figure 3B-G**). This phenomenon is likely due to the presence of cholesterol in the PM 45. We, therefore, assessed the distribution of cholesterol following Etn treatment and found that membrane cholesterol distribution was impaired (**Figure S2B-C**). Profound cholesterol internalization was also observed in PCa xenografts in Etn-treated mice (**Figure S2D**), suggesting that Etn decreases membrane fluidity. Collectively, these results indicate that Etn alters membrane dynamics and fluidity in PCa cells.

### Etn lowers the PE/GPE ratio and inhibits PCa progression

**Figure 4:**
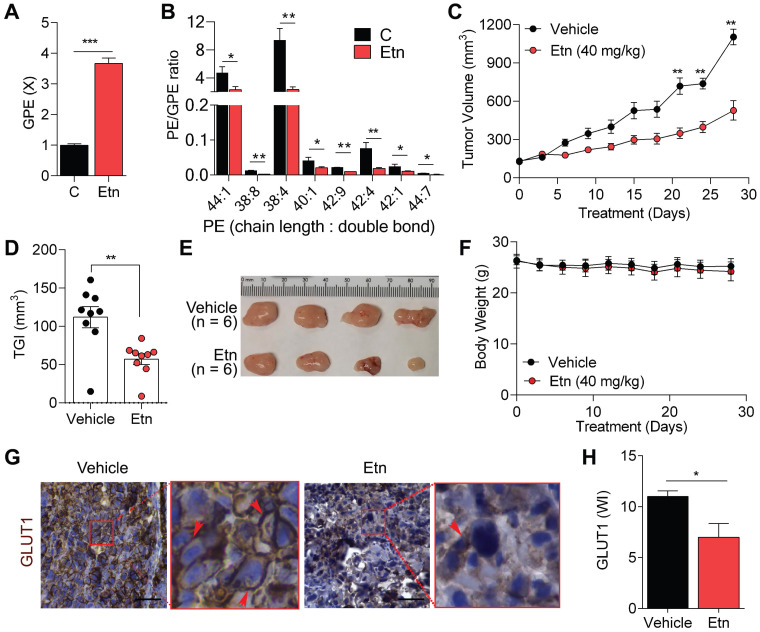
** Etn-induced PE/GPE ratio alterations inhibit tumor progression. (A)** Bar graph showing the levels of GPE in control and Etn-treated PCa cells. **(B)** Quantification of PE and GPE levels using lipidomics and metabolomics analyses to assess the PE/GPE ratio in Etn-treated and untreated PCa cells. **(C-F)** Changes in tumor volume **(C)**, tumor growth inhibition **(D)**, and representative images **(E)** of PC3 xenografts, as well as body weights **(F)** of mice treated with vehicle and Etn for 28 days. **(G-H)** GLUT1 expression in PC3 xenografts of vehicle and Etn-treated mice; representative images **(G)** and quantification data **(H)** are shown. Red arrows indicate membrane staining. C = control (untreated PCa cells). Bars indicate mean ± SEM. Unpaired two-tailed Student's *t*-test with Welch's correction was performed to determine the statistical significance (*P < 0.05, **P < 0.005, ***P < 0.0005). The scale bar represents 10 µm.

Etn is a PE precursor 46, and abnormal PE levels have been shown to modulate membrane structure and dynamics 47. Here, we investigated the effects of Etn treatment on PE levels. Etn treatment increased PE levels (**Figure S2A**), in line with the altered membrane dynamics in PCa cells upon Etn treatment. The excess PE in cells is often deacylated and converted to its phosphodiester form, glycerophosphoethanolamine (GPE) 27, 48. We found a significant increase in GPE levels (**Figure 4A**) and a substantial decrease in the PE/GPE ratio after Etn treatment in PCa cells (**Figure 4B**). The PC/GPC ratio is often used to monitor cancer treatment response and tumor progression 23-25. Here, we sought to determine if the Etn-induced decrease in PE/GPE ratio could similarly be used to monitor tumor progression. We observed a significant reduction in tumor volume (**Figure 4C**) and a ~50% tumor growth inhibition (**Figure 4D-E**) with no significant reduction of body weight (**Figure 4F**) in our xenograft mouse model. These results indicate that, akin to PC/GPC, the PE/GPE ratio can potentially serve as a reliable measure of tumor progression upon Etn treatment.

### Etn disrupts mitochondrial structure and function and increases lipolysis

**Figure 5:**
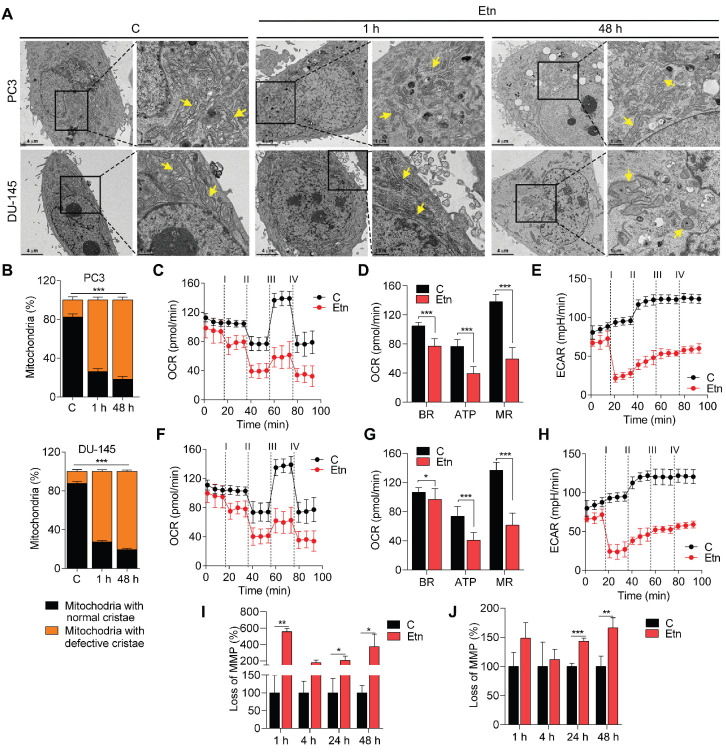
** Disrupted mitochondrial structure and function after Etn treatment. (A-B)** Representative TEM images **(A)** and quantification **(B)** of the mitochondria in untreated and Etn-treated PCa cells. Yellow arrows indicate mitochondria. **(C-F)** Etn reduced OCR in PC3 **(C)** and DU-145 **(F)** cells; i-iv denotes treatment with Etn, oligomycin, FCCP, and rotenone/antimycin, respectively. **(D-G)** Bar graphs showing BR, ATP, and MR levels in control and Etn-treated PC3 **(D)** and DU-145 **(G)** cells. **(E-H)** Etn reduced ECAR in PC3 **(E)** and DU-145 **(H)** cells; i-iv denotes treatment with Etn, oligomycin, FCCP, and rotenone/antimycin, respectively. **(I-J)** Bar graphs showing the percentage of cells with loss of MMP in untreated and Etn-treated PC3 **(I)** and DU-145 **(J)** cells. C = control (untreated PCa cells). Bars indicate mean ± SEM. Unpaired two-tailed Student's *t*-test with Welch's correction was used to determine the statistical significance (*P < 0.05, **P < 0.005, ***P < 0.0005). Scale bars indicate 4 µm and 1 µm.

PE lipids are abundant in mitochondrial membranes, with relatively higher accumulation in the IM 49, 50. The mitochondrial structure and function are primarily influenced by the unique phospholipid composition of mitochondrial membranes 51. To explore the effect of Etn-induced alteration of PE levels on mitochondrial structure, we examined the ultrastructure of mitochondria in Etn-treated PCa cells using TEM (**Figure 5A**). Etn-treated PCa cells exhibited mitochondrial cristae disruption (**Figure 5B**), appearing within 1 h of Etn treatment. This disruption was significantly increased after 48 h of treatment. Recent findings suggest that the mitochondrial structure is linked to cellular bioenergetics 47 and that PE lipids are required for oxidative phosphorylation 49, 50. Therefore, we evaluated the effect of Etn on the mitochondrial function by quantifying real-time OCR and ECAR, which indicate the levels of mitochondrial respiration or oxidative phosphorylation and glycolysis, respectively 28. In both PCa cell lines, Etn treatment significantly reduced OCR (**Figure 5C, F**) and ECAR (**Figure 5E, H**). Furthermore, basal respiration, ATP production, and maximal respiration were reduced after Etn treatment (**Figure 5D, G**), suggesting an impaired mitochondrial function. These effects of Etn treatment on mitochondria are potentially correlated with reduced glucose uptake due to impaired GLUT1 trafficking.

Mitochondrial membrane potential (MMP, ΔΨm) is another measure of mitochondrial function 52. Asymmetric transmembrane distribution of lipid molecules, especially PC and PE, is crucial for maintaining MMP 53, 54. We, therefore, evaluated the effects of Etn treatment on MMP and membrane fluidity. We observed a significant loss of MMP (**Figure 5I-J**) in PCa cells treated with Etn for 24 and 48 h. Altogether, these data strongly suggest that the increase in PE levels in response to Etn treatment significantly impairs mitochondrial structure and function, leading to metabolic stress in PCa. Cells with mitochondrial dysfunction resort to alternative sources of energy, such as LDs, composed of neutral saturated lipids and cholesterol esters. LDs serve as vital hubs of cellular metabolism and energy homeostasis in cancer cells, and their biogenesis and degradation are tightly coupled to cellular metabolism 55. High LD density has been associated with a higher cell proliferation rate and poor prognosis in various cancers, including PCa 56, 57. Here, we investigated the effects of Etn on LD and observed a significant time-dependent reduction in the number of LDs upon Etn treatment (**Figure S3A-B**). We also investigated the fate of LDs that were degraded after Etn treatment by measuring the concentration of extracellular glycerol as an indicator of lipolysis 58. A higher glycerol concentration was observed in the cell culture media of Etn-treated PCa cells (**Figure S3C-D**) compared with that of untreated cells, suggesting that the LD shrinkage or degradation after Etn treatment is likely due to increased lipolysis. These findings indicate that LDs, which function as a buffer to prevent lipotoxic damage to mitochondria, are utilized by PCa cells as an alternative energy source in response to Etn-induced mitochondrial dysfunction and metabolic stress.

### Etn-induced metabolic stress promotes autophagy in PCa cells

**Figure 6:**
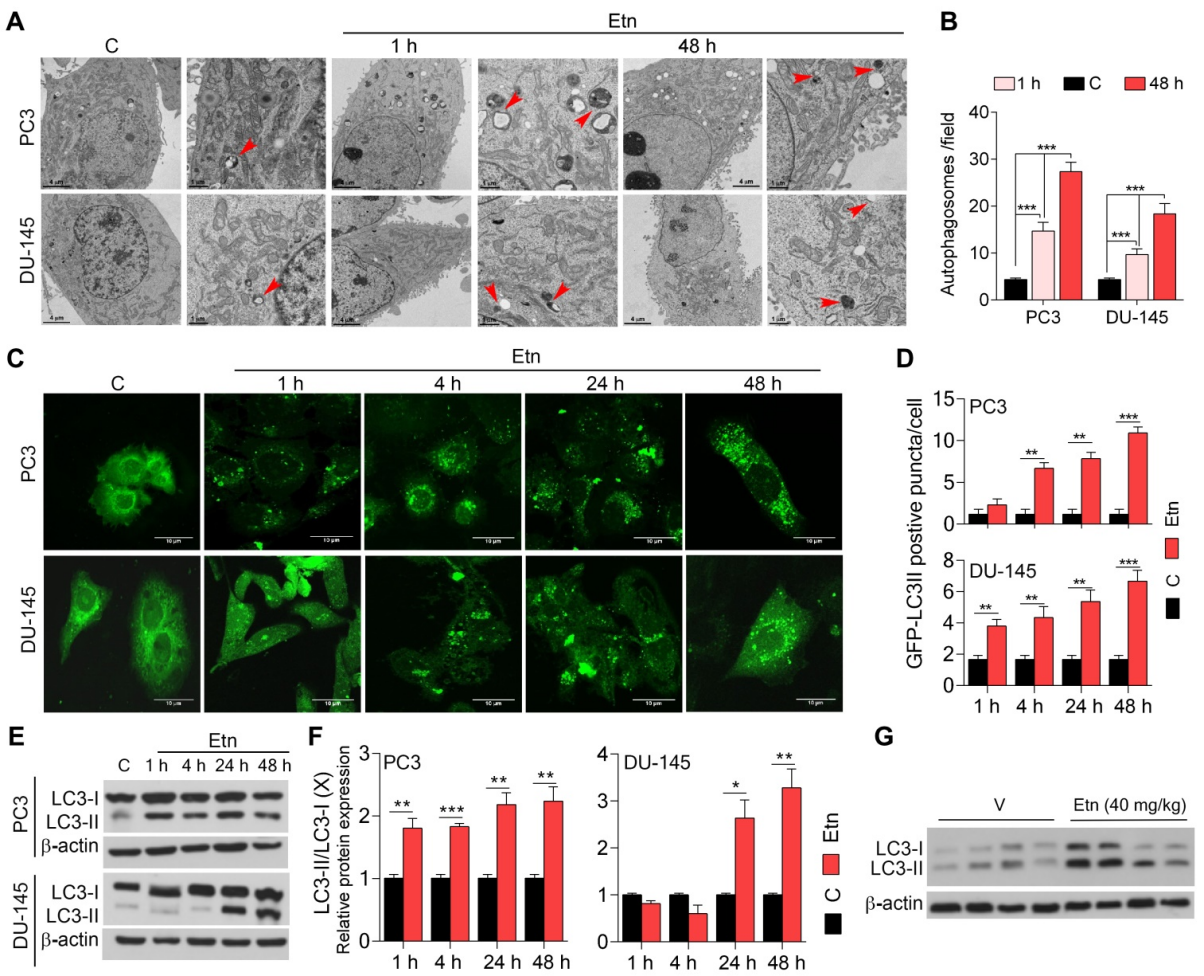
**Etn treatment induces autophagy in PCa. (A-B)** Representative TEM images **(A)** and quantification graphs **(B)** of Etn-treated and untreated PCa cells. Red arrows indicate double-membraned autophagosomes. **(C-D)** Representative immunofluorescence staining of LC3B **(C)** and its quantification **(D)**, showing LC3-II-positive puncta in PCa cells. **(E)** Immunoblot showing the levels of LC3-I and LC3-II in Etn-treated and untreated PCa cells at different time points. **(F)** LC3-II/ LC3-I ratio in PC3 and DU-145 cells. **(G)** Immunoblots showing the conversion of LC3-I to LC3-II upon Etn treatment in mice bearing PC3 xenografts. Unpaired two-tailed Student's *t*-test with Welch's correction was used to determine the statistical significance (*P < 0.05, **P < 0.005, ***P < 0.0005). C = control (untreated PCa cells). Scale bars indicate 4 µm and 1 µm for TEM images **(A)** and 10 µm for the IF images **(C)**.

Under physiological conditions, autophagy occurs at a low level. However, in response to stress, such as deprivation of nutrients, oxidative stress, and accumulation of damaged organelles, autophagy is induced to sustain cellular homeostasis and energy requirements and facilitate cell survival 59-62. TEM revealed the presence of double-membrane autophagosomes after 1 h of Etn treatment (**Figure 6A**). In contrast, untreated control cells contained relatively low numbers of autophagosomes (**Figure 6B**), suggesting that Etn-induced metabolic stress may promote autophagy in PCa cells. We employed a fluorescence cell-based assay to further investigate this phenomenon using an LC3-GFP reporter and immunoblotting for LC3. LC3 is an autophagosome membrane marker, and its cleavage yields two fragments, LC3-I and LC3-II 63; an increase in the LC3II/LC3-I ratio indicates autophagy induction 64. Untreated PCa cells exhibited low levels of LC3-GFP puncta (**Figure 6C**), suggesting a low autophagy activity. However, Etn treatment induced a significant increase (~3-6-fold) in LC3-GFP puncta (**Figure 6C-D**) in a concentration and time-dependent manner, indicating autophagy activation. Consistent with this, we found a significant decrease in LC3-I levels and an increase in LC3-II levels in response to Etn treatment (**Figure 6E-F**). The LC3-II/LC3-I ratio was also increased in Etn-treated PCa cells, confirming autophagy induction. Immunoblots of PC3 xenograft tumors further confirmed autophagy induction in response to Etn treatment (**Figure 6G and Figure S4A**).

We also evaluated the effects of Etn on LC3 protein levels after treatment with the autophagy inhibitors 3-methyl adenine (3-MA) (early stage) and chloroquine (CQ) (late-stage), and the autophagy inducer RAD001. PC3 cells treated with CQ+Etn exhibited the highest autophagy levels, while in DU-145 cells, Etn treatment alone was the most potent in inducing autophagy (**Figure S4B**). As expected, treatment with the autophagy inhibitors 3-MA and CQ significantly altered autophagy levels by affecting the formation and maturation of autophagosomes to autolysosomes. Surprisingly, treatment with Etn combined with 3-MA or CQ significantly increased the overall autophagy levels (**Figure S4B**). Overall, these data strongly suggest that Etn-induced metabolic stress promotes autophagy in PCa cells, which likely acts as a mechanism used by cells to overcome metabolic reprogramming and stress.

### Etn-mediated metabolic stress induces apoptosis in PCa cells

**Figure 7:**
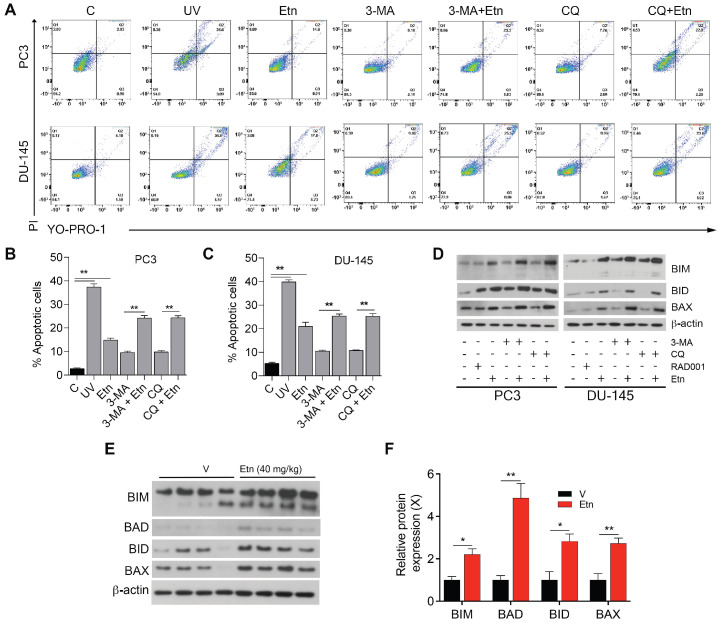
** Etn treatment induces apoptosis in PCa cells. (A-C)** Representative dot plots **(A)** and quantification of live and apoptotic PC3 **(B)** and DU-145 **(C)** cells in the presence of autophagic inhibitors or inducers. Q1: necrotic cells, Q2: late apoptotic cells, Q3: early apoptotic cells, and Q4: live cells. **(D)** Immunoblot of various apoptotic markers in PCa cells with and without Etn treatment and in the presence of autophagic inhibitors. **(E-F)** Immunoblots representing various apoptotic markers **(E)** and quantification bar graphs **(F)** in PC3 xenografts. Unpaired two-tailed Student's *t*-test with Welch's correction was used to determine the statistical significance (*P < 0.05, **P < 0.005, ***P < 0.0005). C = control (untreated PCa cells); PI: propidium iodide; UV: ultraviolet.

Autophagy acts as a double-edged sword; depending on the context, it can have either pro-survival (oncogenic) or pro-death (tumor-suppressive) effects 63, 65, 66. To determine whether Etn-induced autophagy is a pro-survival or pro-death mechanism, we assessed the percentage of apoptotic cells after treatment with autophagy inhibitors (3-MA and CQ) alone or in combination with Etn. The percentage of apoptotic cells in PCa cells treated with autophagy inhibitors alone was slightly higher than in untreated control cells. The combination of autophagy inhibitors with Etn significantly increased the percentage of apoptotic cells (**Figure 7A-C**). These results suggest that Etn enhances apoptosis in the presence of autophagy inhibitors, which likely inhibit the pro-survival mechanism activated by the Etn-induced energy deprivation and metabolic stress. The increase in the levels of pro-apoptotic markers was more prominent in PCa cells treated with autophagy inhibitors combined with Etn than in cells treated with Etn alone (**Figure 7D and Figure S4C-H**). An upregulation of pro-apoptotic markers was also evident in PC3 xenografts in mice treated with Etn compared with those in mice treated with the vehicle (**Figure 7E-F**). These results are consistent with our previous findings 6 and confirm that autophagy acts as an intermediary link between metabolic stress and apoptosis.

## Discussion

Maintaining metabolic homeostasis in response to stress is fundamental to propagating cancer cells and rampant cancer cell proliferation 67. Considering the crucial role of metabolic reprogramming in various cancer types, the administration of metabolism-modulating agents has emerged as a promising anticancer strategy. Multiple compounds have been shown to selectively target metabolic enzymes thus far. Metabolic enzymes under clinical investigation as therapeutic targets include glutaminase 1 (GLS1), monocarboxylate transporter 4 (MST4), MST1, and carbonic anhydrase IX (CAIX) 7.

**Figure 8:**
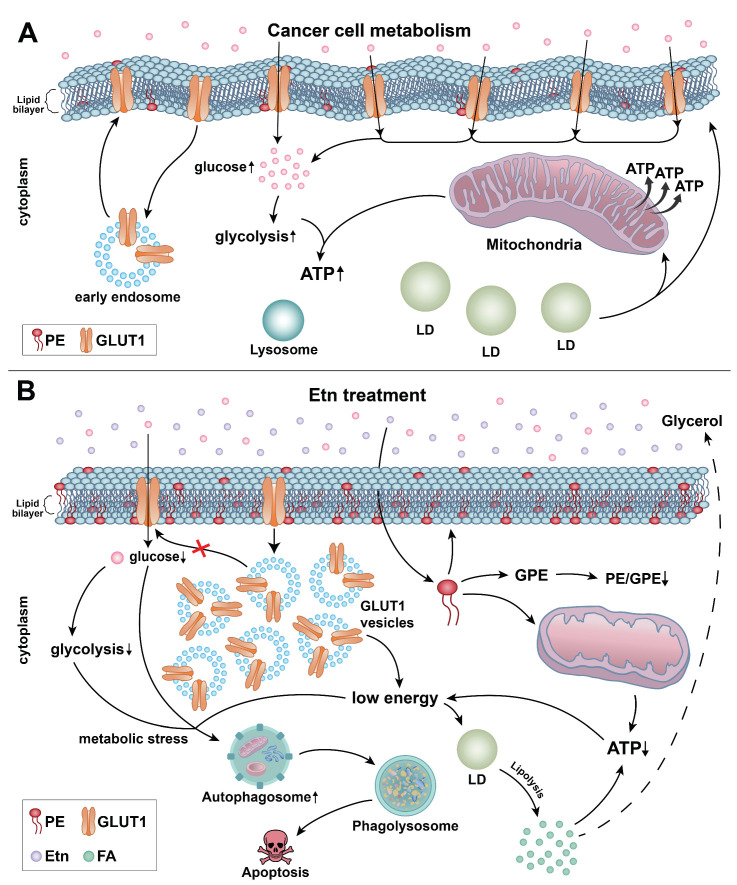
** Schematic representation of the mechanisms underlying Etn-mediated metabolic stress in PCa cells. (A)** The lipid bilayer of the cell membranes of cancer cells is more fluidic or less rigid. Glucose gets transported across the plasma membrane into the cytoplasm by GLUT1. Glucose metabolism through glycolysis (cytoplasm) and in mitochondria provides the energy required for cancer cells to meet their high energy and metabolic demands. **(B)** Etn treatment results in metabolic alterations in PCa cells. Etn lowers the glucose uptake into the cytosol by modulating GLUT1 trafficking, making PCa cells energy-deprived with increased metabolic stress. Etn increases the levels of PE in the membrane, making the membrane less fluidic (or more rigid). PE deacylation into GPE is enhanced, and PE/GPE ratio is decreased. The increased PE levels alter the mitochondrial structure and function and enhance lipolysis, leading to LD shrinkage and generation of fatty acids. The Etn-induced metabolic stress promotes autophagy, subsequently leading to apoptosis in PCa.

We have previously demonstrated that Etn modulates metabolism in PCa cells. Additionally, we showed that Etn reduced PCa tumor volume and weight by 67% and 55%, respectively 6. However, its precise mode of action in lowering PCa tumor burden had not been described. In this study, we explored the mechanisms underlying the Etn-mediated metabolic alterations in PCa and the relevance of Etn-induced metabolic stress in PCa cell survival. Our findings suggest that Etn modulates GLUT1 trafficking, thereby lowering glucose uptake and resulting in nutrient deprivation in PCa cells. Our findings also indicate that conversion of Etn to PE impairs cellular bioenergetics, leading to PCa cell apoptosis (**Figure 8**). In particular, we found that increased PE levels upon Etn treatment could modulate mitochondrial structure and function, thereby inducing metabolic stress. Failure to adapt to the Etn-induced metabolic stress triggers autophagy, leading to apoptosis in PCa cells. Thus, Etn could serve as a promising therapeutic regimen for PCa.

Glucose metabolism is critical for PCa cell proliferation and survival. The increased demand for glucose in PCa results in the upregulation of GLUTs, particularly of GLUT1, which is essential for cancer cell viability 68, 69. Modulating cancer cell metabolism through glucose deprivation has been proposed as an effective approach to eliminate cancer cells 70. Although inhibiting GLUTs can deprive cells of glucose, the non-specific toxicity of GLUT inhibitors has been a hindrance in their clinical development as anticancer agents. Recent crystalization of GLUTs has opened new avenues for the development of novel GLUT-specific inhibitors 71, 72. However, there have been no clinical trials assessing the efficacy and safety of specific GLUT inhibitors thus far. In the present study, we developed an alternative glucose deprivation approach using a lipid-based formulation that effectively modulates GLUT1 trafficking. Our data suggest that Etn impairs GLUT1 recycling by inducing GLUT1 internalization into the cytosol and inhibiting its trafficking back to the PM. By doing so, Etn reduces glucose uptake and induces metabolic stress in PCa cells. The altered membrane dynamics due to PE upregulation are seemingly responsible for the dysregulated GLUT1 trafficking. Phospholipids are crucial components of cell membranes, and their content has been reported to increase during cell transformation and tumor progression. Thus, aberrant phospholipid metabolism is regarded as a metabolic hallmark of cancer 73. Compelling data by Beckonert et al. 74 suggest that the levels of two major phospholipids, PC and PE, are associated with tumor grade in breast cancer. Moreover, a relative decrease in GPC levels has been reported in breast and ovarian cancer cells; hence, a high PC/GPC ratio can indicate tumor progression 19, 75. Similarly, a relative decrease in GPE levels accompanied by an increase in PE/GPE ratio could indicate a high tumor burden. In this study, we found that the PE precursor Etn alters the PE/GPE ratio. Based on our findings in a xenograft mouse model, the use of the PE/GPE ratio as a potential malignancy indicator merits further investigation.

PE is a crucial component of the mitochondrial inner membrane, and changes in PE levels can modulate mitochondrial membrane structure and function. The structural and functional integrity of mitochondria is essential for maintaining cell homeostasis by providing various metabolic intermediates and maintaining a balance between pro-apoptotic and anti-apoptotic proteins 49, 50, 76. Alterations in mitochondrial activity can disrupt metabolic processes, which, in turn, suppress tumorigenesis 9. Moreover, *in vitro* studies in liposomes demonstrated that, along with cholesterol, PE lipids play a vital role in increasing the rigidity of lipid bilayers and maintaining membrane fluidity 77. Consistent with these data, we found that alterations in PE levels after Etn treatment may impact the mitochondrial and cellular bioenergetics of PCa cells. Apart from its crucial role in mitochondrial function, PE is also vital for autophagosome formation to encase cargos destined for degradation in the lysosomes 78-80. Although autophagy has been considered a cell survival mechanism in response to nutrient starvation 81, 82, recent evidence suggests that it can promote apoptotic cell death 81, 83. Our results and previous work by Saxena et al. 6 indicate that Etn-mediated metabolic stress induces autophagy in a failed attempt to survive, which in turn leads to apoptotic cell death in PCa cells.

In summary, we report that Etn remodels metabolic activities in PCa cells at various levels and disrupts nutrient and energy balance. Notably, Etn effectively modulates GLUT1 recycling and depletes cellular glucose levels. To the best of our knowledge, this is the first study to report that along with the PC/GPC ratio, the PE/GPE ratio could also serve as a robust indicator of cancer progression. Etn-mediated enhanced lipolysis decreases LD density in the cell cytoplasm, as evidenced by the increased glycerol export to the extracellular environment. Etn-induced metabolic stress promotes autophagy and apoptotic cell death, suppressing tumor progression. Thus, Etn should be further investigated as a potential nontoxic anti-PCa drug, which primarily exerts its antitumor effects by modulating cancer cell metabolism. Future metabolomics studies are required to identify the distinct metabolomic pathways affected by Etn and better understand its mechanisms of action. As the metabolic interplay in the tumor microenvironment is heterogeneous, combining Etn with other chemotherapeutic agents may further advance treatment efficacy.

## Supplementary Material

Supplementary figures.

## References

[1] Santos CR, Schulze A (2012). Lipid metabolism in cancer. FEBS J.

[2] Drugs on demand Nature. 2015; 527: 410.

[3] Ledford H (2015). Brazilian courts tussle over unproven cancer treatment. Nature.

[4] Ferreira AK, Meneguelo R, Pereira A, Filho OM, Chierice GO, Maria DA (2013). Synthetic phosphoethanolamine induces cell cycle arrest and apoptosis in human breast cancer MCF-7 cells through the mitochondrial pathway. Biomed Pharmacother.

[5] Ferreira AK, Santana-Lemos BA, Rego EM, Filho OM, Chierice GO, Maria DA (2013). Synthetic phosphoethanolamine has in vitro and in vivo anti-leukemia effects. Br J Cancer.

[6] Saxena R, Yang C, Rao M, Turaga RC, Garlapati C, Gundala SR (2017). Preclinical Development of a Nontoxic Oral Formulation of Monoethanolamine, a Lipid Precursor, for Prostate Cancer Treatment. Clin Cancer Res.

[7] Phan LM, Yeung SC, Lee MH (2014). Cancer metabolic reprogramming: importance, main features, and potentials for precise targeted anti-cancer therapies. Cancer Biol Med.

[8] Hanahan D, Weinberg RA (2011). Hallmarks of cancer: the next generation. Cell.

[9] DeBerardinis RJ, Chandel NS (2016). Fundamentals of cancer metabolism. Sci Adv.

[10] Fadaka A, Ajiboye B, Ojo O, Adewale O, Olayide I, Emuowhochere R (2017). Biology of glucose metabolization in cancer cells. Journal of Oncological Sciences.

[11] Macheda ML, Rogers S, Best JD (2005). Molecular and cellular regulation of glucose transporter (GLUT) proteins in cancer. J Cell Physiol.

[12] Wallace DC (2012). Mitochondria and cancer. Nat Rev Cancer.

[13] Weinberg F, Hamanaka R, Wheaton WW, Weinberg S, Joseph J, Lopez M (2010). Mitochondrial metabolism and ROS generation are essential for Kras-mediated tumorigenicity. Proc Natl Acad Sci U S A.

[14] Cheng C, Geng F, Cheng X, Guo D (2018). Lipid metabolism reprogramming and its potential targets in cancer. Cancer Commun (Lond).

[15] Menendez JA, Lupu R (2007). Fatty acid synthase and the lipogenic phenotype in cancer pathogenesis. Nat Rev Cancer.

[16] Henne WM, Reese ML, Goodman JM (2018). The assembly of lipid droplets and their roles in challenged cells. EMBO J.

[17] Casares D, Escriba PV, Rossello CA (2019). Membrane Lipid Composition: Effect on Membrane and Organelle Structure, Function and Compartmentalization and Therapeutic Avenues. Int J Mol Sci.

[18] Patel D, Witt SN (2017). Ethanolamine and Phosphatidylethanolamine: Partners in Health and Disease. Oxid Med Cell Longev.

[19] Aboagye EO, Bhujwalla ZM (1999). Malignant transformation alters membrane choline phospholipid metabolism of human mammary epithelial cells. Cancer Res.

[20] van der Kemp WJ, Stehouwer BL, Runge JH, Wijnen JP, Nederveen AJ, Luijten PR (2016). Glycerophosphocholine and Glycerophosphoethanolamine Are Not the Main Sources of the In Vivo (31)P MRS Phosphodiester Signals from Healthy Fibroglandular Breast Tissue at 7 T. Front Oncol.

[21] Dobrzynska I, Szachowicz-Petelska B, Darewicz B, Figaszewski ZA (2015). Characterization of human bladder cell membrane during cancer transformation. J Membr Biol.

[22] Szachowicz-Petelska B, Dobrzynska I, Skrodzka M, Darewicz B, Figaszewski ZA, Kudelski J (2013). Phospholipid composition and electric charge in healthy and cancerous parts of human kidneys. J Membr Biol.

[23] Glunde K, Jie C, Bhujwalla ZM (2006). Mechanisms of indomethacin-induced alterations in the choline phospholipid metabolism of breast cancer cells. Neoplasia.

[24] Cheng M, Rizwan A, Jiang L, Bhujwalla ZM, Glunde K (2017). Molecular Effects of Doxorubicin on Choline Metabolism in Breast Cancer. Neoplasia.

[25] Iorio E, Ricci A, Bagnoli M, Pisanu ME, Castellano G, Di Vito M (2010). Activation of phosphatidylcholine cycle enzymes in human epithelial ovarian cancer cells. Cancer Res.

[26] Esko JD, Nishijima M, Raetz CR (1982). Animal cells dependent on exogenous phosphatidylcholine for membrane biogenesis. Proc Natl Acad Sci U S A.

[27] Stalberg K, Neal AC, Ronne H, Stahl U (2008). Identification of a novel GPCAT activity and a new pathway for phosphatidylcholine biosynthesis in S. cerevisiae. J Lipid Res.

[28] Plitzko B, Loesgen S (2018). Measurement of Oxygen Consumption Rate (OCR) and Extracellular Acidification Rate (ECAR) in Culture Cells for Assessment of the Energy Metabolism. Bio Protoc.

[29] Panikkanvalappil SR, Garlapati C, Hooshmand N, Aneja R, El-Sayed MA (2019). Monitoring the dynamics of hemeoxygenase-1 activation in head and neck cancer cells in real-time using plasmonically enhanced Raman spectroscopy. Chem Sci.

[30] Owen DM, Rentero C, Magenau A, Abu-Siniyeh A, Gaus K (2011). Quantitative imaging of membrane lipid order in cells and organisms. Nat Protoc.

[31] Aron M, Browning R, Carugo D, Sezgin E, Bernardino de la Serna J, Eggeling C (2017). Spectral imaging toolbox: segmentation, hyperstack reconstruction, and batch processing of spectral images for the determination of cell and model membrane lipid order. BMC Bioinformatics.

[32] Breil C, Abert Vian M, Zemb T, Kunz W, Chemat F (2017). "Bligh and Dyer" and Folch Methods for Solid-Liquid-Liquid Extraction of Lipids from Microorganisms. Comprehension of Solvatation Mechanisms and towards Substitution with Alternative Solvents. Int J Mol Sci.

[33] Ogden A, Garlapati C, Li XX, Turaga RC, Oprea-Ilies G, Wright N (2017). Multi-institutional study of nuclear KIFC1 as a biomarker of poor prognosis in African American women with triple-negative breast cancer. Sci Rep.

[34] Lin X, Xiao Z, Chen T, Liang SH, Guo H (2020). Glucose Metabolism on Tumor Plasticity, Diagnosis, and Treatment. Front Oncol.

[35] Ancey PB, Contat C, Meylan E (2018). Glucose transporters in cancer - from tumor cells to the tumor microenvironment. FEBS J.

[36] Reinicke K, Sotomayor P, Cisterna P, Delgado C, Nualart F, Godoy A (2012). Cellular distribution of Glut-1 and Glut-5 in benign and malignant human prostate tissue. J Cell Biochem.

[37] Chandrashekar DS, Bashel B, Balasubramanya SAH, Creighton CJ, Ponce-Rodriguez I, Chakravarthi B (2017). UALCAN: A Portal for Facilitating Tumor Subgroup Gene Expression and Survival Analyses. Neoplasia.

[38] Tanner LB, Goglia AG, Wei MH, Sehgal T, Parsons LR, Park JO (2018). Four Key Steps Control Glycolytic Flux in Mammalian Cells. Cell Syst.

[39] Zambrano A, Molt M, Uribe E, Salas M (2019). Glut 1 in Cancer Cells and the Inhibitory Action of Resveratrol as A Potential Therapeutic Strategy. Int J Mol Sci.

[40] Lee EE, Ma J, Sacharidou A, Mi W, Salato VK, Nguyen N (2015). A Protein Kinase C Phosphorylation Motif in GLUT1 Affects Glucose Transport and is Mutated in GLUT1 Deficiency Syndrome. Mol Cell.

[41] Perona JS (2017). Membrane lipid alterations in the metabolic syndrome and the role of dietary oils. Biochim Biophys Acta Biomembr.

[42] Habegger KM, Hoffman NJ, Ridenour CM, Brozinick JT, Elmendorf JS (2012). AMPK enhances insulin-stimulated GLUT4 regulation via lowering membrane cholesterol. Endocrinology.

[43] Yuli I, Wilbrandt W, Shinitzky M (1981). Glucose transport through cell membranes of modified lipid fluidity. Biochemistry.

[44] Melcrova A, Pokorna S, Pullanchery S, Kohagen M, Jurkiewicz P, Hof M (2016). The complex nature of calcium cation interactions with phospholipid bilayers. Sci Rep.

[45] Sok M, Sentjurc M, Schara M, Stare J, Rott T (2002). Cell membrane fluidity and prognosis of lung cancer. Ann Thorac Surg.

[46] Kennedy EP, Weiss SB (1956). The function of cytidine coenzymes in the biosynthesis of phospholipides. J Biol Chem.

[47] Cogliati S, Enriquez JA, Scorrano L (2016). Mitochondrial Cristae: Where Beauty Meets Functionality. Trends Biochem Sci.

[48] Mineta S, Murayama K, Sugimori D (2015). Characterization of glycerophosphoethanolamine ethanolaminephosphodiesterase from Streptomyces sanglieri. J Biosci Bioeng.

[49] Daum G (1985). Lipids of mitochondria. Biochim Biophys Acta.

[50] van Meer G, Voelker DR, Feigenson GW (2008). Membrane lipids: where they are and how they behave. Nat Rev Mol Cell Biol.

[51] Basu Ball W, Neff JK, Gohil VM (2018). The role of nonbilayer phospholipids in mitochondrial structure and function. FEBS Lett.

[52] Zorova LD, Popkov VA, Plotnikov EY, Silachev DN, Pevzner IB, Jankauskas SS (2018). Mitochondrial membrane potential. Anal Biochem.

[53] Gurtovenko AA, Vattulainen I (2008). Membrane potential and electrostatics of phospholipid bilayers with asymmetric transmembrane distribution of anionic lipids. J Phys Chem B.

[54] Tan LT, Chan KG, Pusparajah P, Lee WL, Chuah LH, Khan TM (2017). Targeting Membrane Lipid a Potential Cancer Cure?. Front Pharmacol.

[55] Olzmann JA, Carvalho P (2019). Dynamics and functions of lipid droplets. Nat Rev Mol Cell Biol.

[56] Biswas S, Lunec J, Bartlett K (2012). Non-glucose metabolism in cancer cells-is it all in the fat?. Cancer Metastasis Rev.

[57] Mitra R, Le TT, Gorjala P, Goodman OB Jr (2017). Positive regulation of prostate cancer cell growth by lipid droplet forming and processing enzymes DGAT1 and ABHD5. BMC Cancer.

[58] Schweiger M, Eichmann TO, Taschler U, Zimmermann R, Zechner R, Lass A (2014). Measurement of lipolysis. Methods Enzymol.

[59] Lum JJ, Bauer DE, Kong M, Harris MH, Li C, Lindsten T (2005). Growth factor regulation of autophagy and cell survival in the absence of apoptosis. Cell.

[60] Onodera J, Ohsumi Y (2005). Autophagy is required for maintenance of amino acid levels and protein synthesis under nitrogen starvation. J Biol Chem.

[61] Sun F, Xu X, Wang X, Zhang B (2016). Regulation of autophagy by Ca(2). Tumour Biol.

[62] Harr MW, Distelhorst CW (2010). Apoptosis and autophagy: decoding calcium signals that mediate life or death. Cold Spring Harb Perspect Biol.

[63] Codogno P, Meijer AJ (2005). Autophagy and signaling: their role in cell survival and cell death. Cell Death Differ.

[64] Kabeya Y, Mizushima N, Yamamoto A, Oshitani-Okamoto S, Ohsumi Y, Yoshimori T (2004). LC3, GABARAP and GATE16 localize to autophagosomal membrane depending on form-II formation. J Cell Sci.

[65] Inguscio V, Panzarini E, Dini L (2012). Autophagy Contributes to the Death/Survival Balance in Cancer PhotoDynamic Therapy. Cells.

[66] Liu B, Wen X, Cheng Y (2013). Survival or death: disequilibrating the oncogenic and tumor suppressive autophagy in cancer. Cell Death Dis.

[67] Cairns RA, Harris IS, Mak TW (2011). Regulation of cancer cell metabolism. Nature Rev Cancer.

[68] Cutruzzola F, Giardina G, Marani M, Macone A, Paiardini A, Rinaldo S (2017). Glucose Metabolism in the Progression of Prostate Cancer. Front Physiol.

[69] Kapoor K, Finer-Moore JS, Pedersen BP, Caboni L, Waight A, Hillig RC (2016). Mechanism of inhibition of human glucose transporter GLUT1 is conserved between cytochalasin B and phenylalanine amides. Proc Natl Acad Sci U S A.

[70] El Mjiyad N, Caro-Maldonado A, Ramirez-Peinado S, Munoz-Pinedo C (2011). Sugar-free approaches to cancer cell killing. Oncogene.

[71] Wood TE, Dalili S, Simpson CD, Hurren R, Mao X, Saiz FS (2008). A novel inhibitor of glucose uptake sensitizes cells to FAS-induced cell death. Mol Cancer Ther.

[72] Gonzalez-Menendez P, Hevia D, Mayo JC, Sainz RM (2018). The dark side of glucose transporters in prostate cancer: Are they a new feature to characterize carcinomas?. Int J Cancer.

[73] Cheng M, Bhujwalla ZM, Glunde K (2016). Targeting Phospholipid Metabolism in Cancer. Front Oncol.

[74] Beckonert O, Monnerjahn J, Bonk U, Leibfritz D (2003). Visualizing metabolic changes in breast-cancer tissue using 1H-NMR spectroscopy and self-organizing maps. NMR Biomed.

[75] Iorio E, Mezzanzanica D, Alberti P, Spadaro F, Ramoni C, D'Ascenzo S (2005). Alterations of choline phospholipid metabolism in ovarian tumor progression. Cancer Res.

[76] Calzada E, Avery E, Sam PN, Modak A, Wang C, McCaffery JM (2019). Phosphatidylethanolamine made in the inner mitochondrial membrane is essential for yeast cytochrome bc1 complex function. Nat Commun.

[77] Dawaliby R, Trubbia C, Delporte C, Noyon C, Ruysschaert JM, Van Antwerpen P (2016). Phosphatidylethanolamine Is a Key Regulator of Membrane Fluidity in Eukaryotic Cells. J Biol Chem.

[78] van der Veen JN, Kennelly JP, Wan S, Vance JE, Vance DE, Jacobs RL (2017). The critical role of phosphatidylcholine and phosphatidylethanolamine metabolism in health and disease. Biochim Biophys Acta Biomembr.

[79] Rockenfeller P, Koska M, Pietrocola F, Minois N, Knittelfelder O, Sica V (2015). Phosphatidylethanolamine positively regulates autophagy and longevity. Cell Death Differ.

[80] Andrejeva G, Gowan S, Lin G, Wong Te Fong AL, Shamsaei E, Parkes HG (2020). De novo phosphatidylcholine synthesis is required for autophagosome membrane formation and maintenance during autophagy. Autophagy.

[81] Marino G, Niso-Santano M, Baehrecke EH, Kroemer G (2014). Self-consumption: the interplay of autophagy and apoptosis. Nat Rev Mol Cell Biol.

[82] Kroemer G, Marino G, Levine B (2010). Autophagy and the integrated stress response. Mol Cell.

[83] Maiuri MC, Zalckvar E, Kimchi A, Kroemer G (2007). Self-eating and self-killing: crosstalk between autophagy and apoptosis. Nat Rev Mol Cell Biol.

