# Supplementary material for: Monoethanolamine-induced glucose deprivation promotes apoptosis through metabolic rewiring in prostate cancer
Source: Theranostics. 2021 Aug 27;11(18):9089–106. doi: 10.7150/thno.62724 (PMC8419048; doi:10.7150/thno.62724)
Supplement: Supplementary file 1 — Supplementary figures. [file thnov11p9089s1.pdf]

Supplemental Information

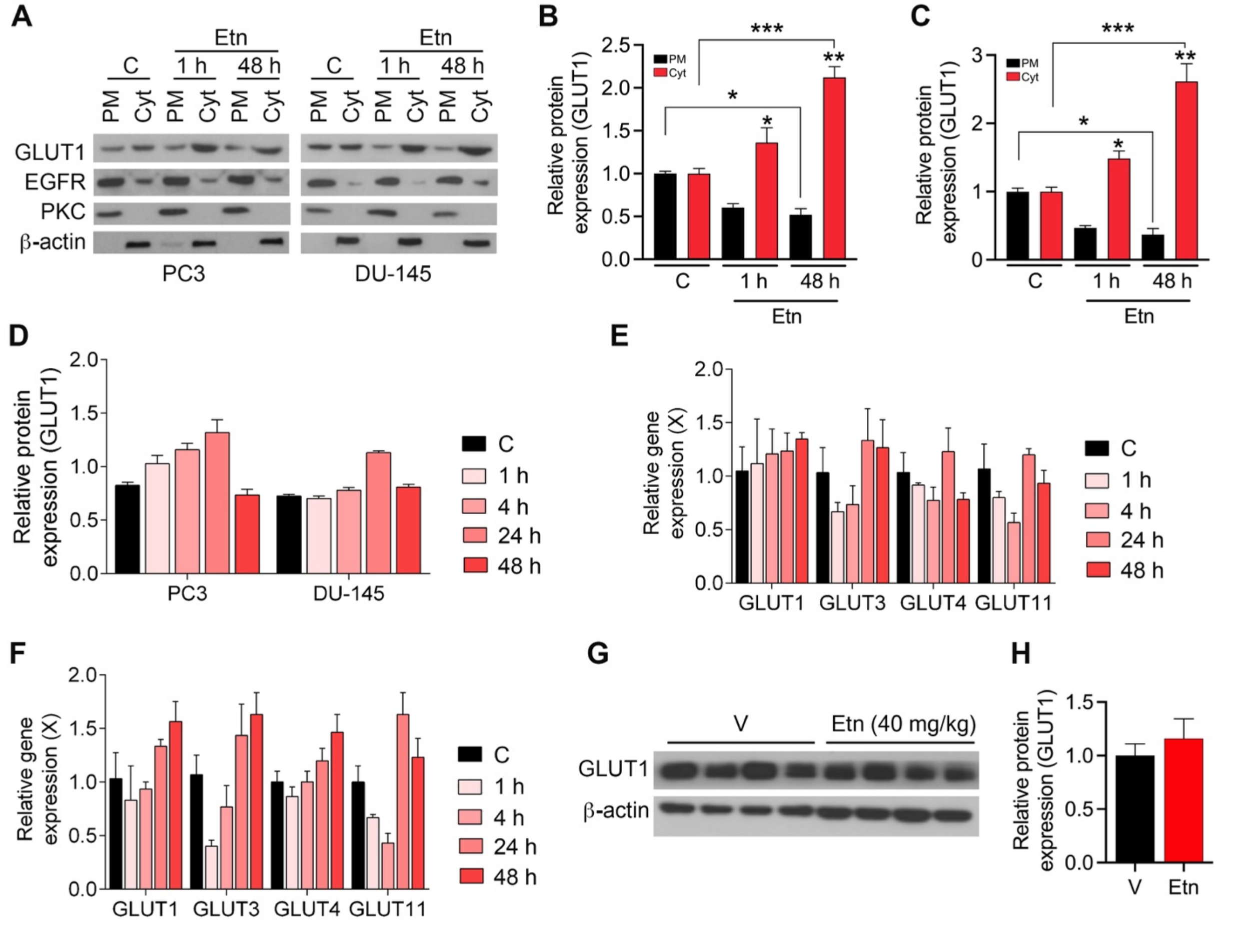

Figure S1. Expression of various GLUTs upon Etn treatment. (A-C) Immunoblots **(A)** and quantification of PC3 **(B)** and DU-145 **(C)** cells showing expression levels of GLUT1 and PKC in the membrane (PM) and cytoplasmic (Cyt) fractions of control and Etn-treated cells. EGFR and β–actin were used as loading control for the membrane and cytoplasmic fractions, respectively. **(D)** Quantification graph of immunoblot shown in **Figure 2E. (E-F)** Relative gene expression of various GLUTs in PC3 **(E)** and DU-145 **(F)** cells with and without Etn treatment at different time points. **(G-H)** Immunoblot **(G)** of GLUT1 in PC3 xenograft and its quantification **(H)**. Bars indicate mean ± SEM. Unpaired two-tailed Student's *t*-test was used to determine the statistical significance.

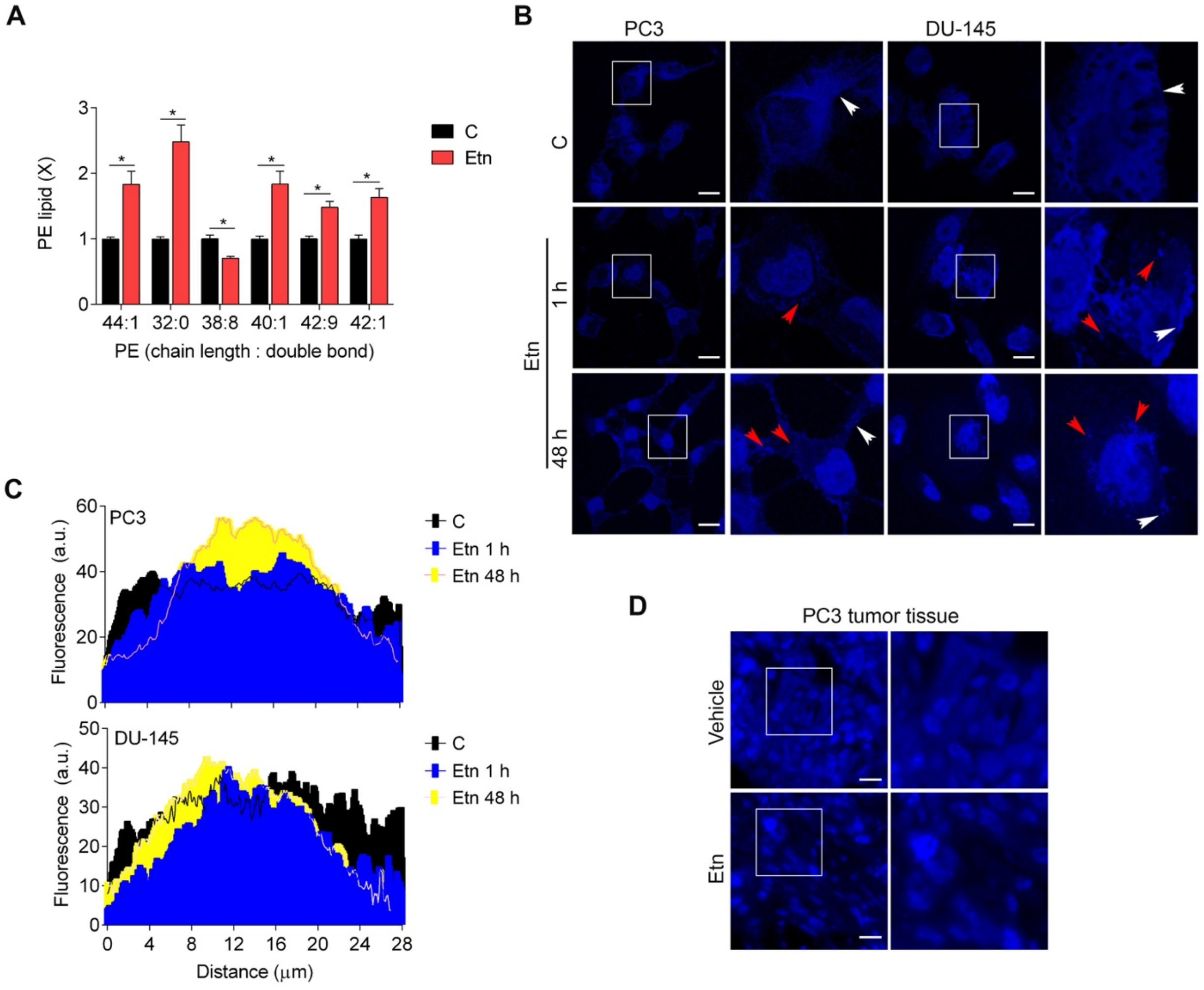

Figure S2. Etn increases PE levels and alters membrane cholesterol levels. (A) Changes in PE levels in Etn-treated and untreated PCa cells. **(B-D)** Immunofluorescence cholesterol staining using filipin in PCa cells **(B)**, quantification **(C)**, in PC3 xenografts **(D)**. Red arrows indicate cytoplasmic cholesterol, and white arrows indicate membrane cholesterol. C = control (untreated PCa cells). Bars indicate the mean ± SEM. Unpaired two-tailed Student's *t-test* with Welch's correction was used to determine the statistical significance (**P* < 0.05, ***P* < 0.005, ****P* < 0.0005). Scale bars represent 10 μm.

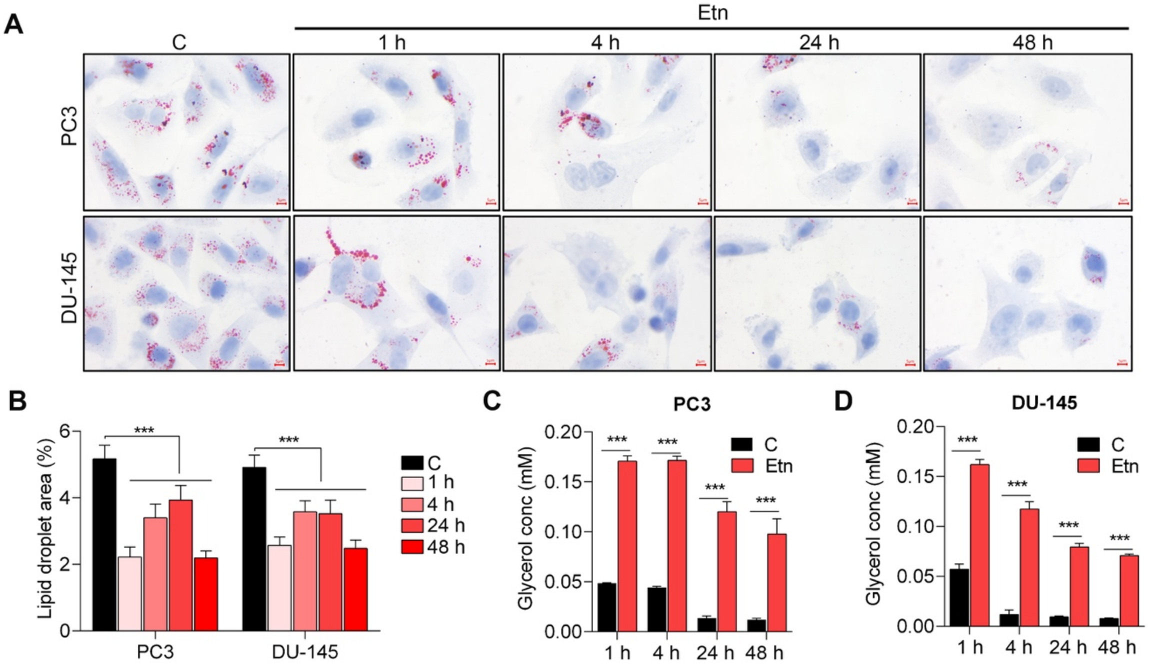

Figure S3. Etn decreases LD density and enhances lipolysis. (A-B) Representative images **(A)** and quantification **(B)** of ORO staining in Etn-treated and untreated PCa cells. ORO (red) represents LDs, while hematoxylin (blue) represents nuclei. 250-300 cells/10 fields were analyzed to determine the percentage LD area. **(C-D)** Bar graphs showing glycerol concentration in Etn-treated and untreated PC3 **(C)** and DU-145 **(D)** cells. Bars indicate mean ± SEM. Unpaired two-tailed Student's *t-test* with Welch's correction was used to determine the statistical significance (*$P < 0.05$, **$P < 0.005$, ***$P < 0.0005$). Scale bars indicate 5 μm; 100x oil objective.

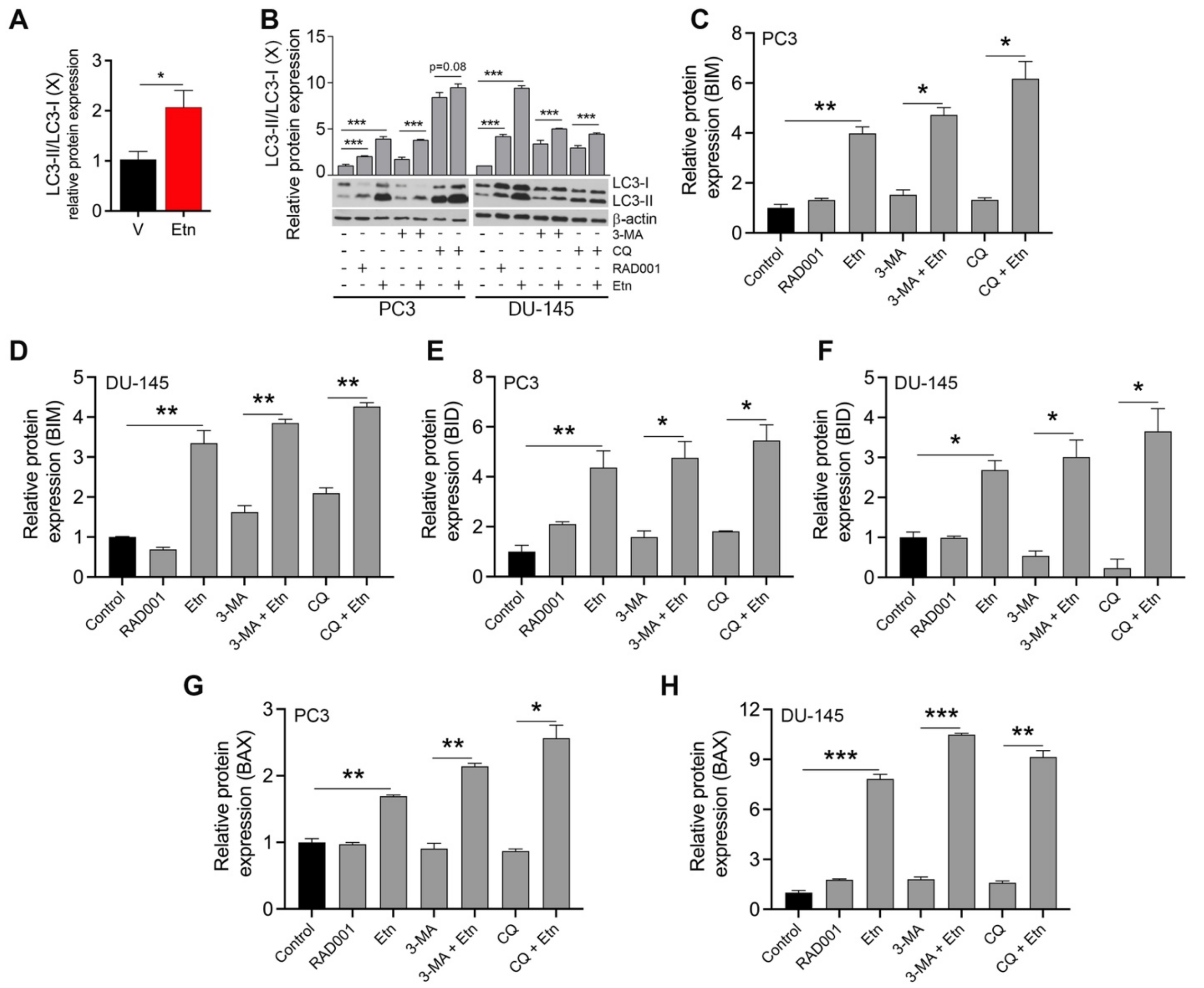

Figure S4. Etn treatment induces autophagy in PCa cells. (A) Quantification of immunoblots shown in **Figure 6G**. **(B)** Immunoblots (bottom) and quantification (top) of LC3-I and LC3-II levels in Etn-treated and untreated PCa cells after treatment with autophagy modulators. **(C-H)** Quantification of BIM, BID, and BAX immunoblots in PC3 **(C, E, G)** and DU-145 **(D, F, H)** cells shown in **Figure 7D**. Bars indicate mean ± SEM. Unpaired two-tailed Student's *t-test* with Welch's correction was used to determine the statistical significance (*$P < 0.05$, **$P < 0.005$, ***$P < 0.0005$). C = control (untreated PCa cells).
